# Sulforaphane Attenuation of Type 2 Diabetes-Induced Aortic Damage Was Associated with the Upregulation of Nrf2 Expression and Function

**DOI:** 10.1155/2014/123963

**Published:** 2014-02-23

**Authors:** Yonggang Wang, Zhiguo Zhang, Wanqing Sun, Yi Tan, Yucheng Liu, Yang Zheng, Quan Liu, Lu Cai, Jian Sun

**Affiliations:** ^1^Cardiovascular Center at the First Hospital of Jilin University, 71 Xinmin Street, Changchun 130021, China; ^2^Kosair Children's Hospital Research Institute, Department of Pediatrics, University of Louisville, Louisville, KY 40202, USA; ^3^The Chinese-American Research Institute, Wenzhou Medical University, Wenzhou 325035, China; ^4^Departments of Radiation Oncology, Pharmacology and Toxicology, University of Louisville, Louisville, KY 40292, USA

## Abstract

Type 2 diabetes mellitus (T2DM) significantly increases risk for vascular complications. Diabetes-induced aorta pathological changes are predominantly attributed to oxidative stress. Nuclear factor E2-related factor-2 (Nrf2) is a transcription factor orchestrating antioxidant and cytoprotective responses to oxidative stress. Sulforaphane protects against oxidative damage by increasing Nrf2 expression and its downstream target genes. Here we explored the protective effect of sulforaphane on T2DM-induced aortic pathogenic changes in C57BL/6J mice which were fed with high-fat diet for 3 months, followed by a treatment with streptozotocin at 100 mg/kg body weight. Diabetic and nondiabetic mice were randomly divided into groups with and without 4-month sulforaphane treatment. Aorta of T2DM mice exhibited significant increases in the wall thickness and structural derangement, along with significant increases in fibrosis (connective tissue growth factor and transforming growth factor), inflammation (tumor necrosis factor-**α** and vascular cell adhesion molecule 1), oxidative/nitrative stress (3-nitrotyrosine and 4-hydroxy-2-nonenal), apoptosis, and cell proliferation. However, these pathological changes were significantly attenuated by sulforaphane treatment that was associated with a significant upregulation of Nrf2 expression and function. These results suggest that sulforaphane is able to upregulate aortic Nrf2 expression and function and to protect the aorta from T2DM-induced pathological changes.

## 1. Introduction

Type 2 diabetes mellitus (T2DM) is a growing public health problem, associated with a substantial burden of morbidity and mortality [1]. Youth with T2DM had higher rates of all complications than nondiabetes subjects, and an overall 6.15-fold increased risk of any vascular disease [2]. Individuals with T2DM have a unique propensity towards microvascular and macrovascular diseases [3]. The macrovascular disorders include atherosclerosis, coronary artery disease, and peripheral vascular diseases. Although application of drugs and changing life style had been widely promoted to control the complications, unfortunately, the preventive of the development and progression of vascular complications in the diabetic patients remains unoptimistic [4]. Therefore, an effective approach to prevent and/or delay the development and progression of diabetic vascular complications urgently needs to be developed.

Impaired endothelial function is considered as a major diabetic vascular alteration, which may be mainly derived from diabetes-induced overexpression of inflammation. Diabetic inflammation in the vascular endothelium leads to continuous infiltration and accumulation of leukocytes at sites of endothelial cell injury. Consistent with our previous studies, vascular inflammatory response was increased in diabetic mice reflect by increased expression of tumor necrosis factor-alpha (TNF-*α*) and vascular cell adhesion molecule 1 (VCAM-1) [5, 6]. It is known that inflammation and oxidative stress are reciprocal cause and outcomes [7]. There was also increasing evidence indicating that the increased production of reactive oxygen and/or nitrogen species (ROS and/or RNS) is the major pathogenic factor responsible for the development and progression of vascular complications in diabetic patients, although several other mechanisms are also proposed [8–10]. Clinical trials with single antioxidants or a few have shown ineffective intervention in diabetic patients [6, 9–11]; therefore, upregulation of multiple endogenous antioxidants may be a better approach for the prevention of diabetic vascular complications. Transcription factor nuclear factor E2-related factor-2 (Nrf2) has been shown to play a pivotal role in cellular preventing against oxidative stress and damage *in vitro* and *in vivo* [12, 13]. Under physiological conditions, Nrf2 generally localizes in the cytoplasm and binds to its inhibitor Kelch-like ECH-associated protein 1 (Keap1) [14]; however, under oxidative or electrophilic stress conditions, Nrf2 detaches from Keap1 and translocates into the nucleus to bind to antioxidant-responsive elements (ARE) in the promoter region of its downstream genes, such as NADPH quinoneoxidoreductase (NQO1), heme oxygenase-1 (HO-1), glutathione S-transferase (GST), superoxide dismutase (SOD), catalase (CAT), and other genes regulating the responses to oxidative stress [15, 16]. The Nrf2-ARE pathway is important in the cellular detoxification, antioxidant, and anti-inflammatory system to protect the cell and tissue from oxidative stress [17, 18]. Therefore, Nrf2 is widely appreciated for its potential prevention of and/or therapy for diabetic vascular complications [5, 19].

Sulforaphane (SFN) is an isothiocyanate that is found in cruciferous vegetables and has a strong cytoprotective function against oxidative stress. This beneficial effect was mediated by its direct interaction with Keap1, resulting in the disruption of Nrf2—Keap1 interaction. Released Nrf2 from Keap1 enters into the nucleus to induce the expression of Nrf2 downstream antioxidant genes [14]. Evidence has confirmed the specific cysteine residues of Keap1 that act as “sensors” to be modified by SFN [20]. SFN as a Nrf2 activator [21] has been reported to prevent oxidative damage [22] and cardiovascular diseases [23]. SFN has garnered particular interests as an indirect antioxidant due to its extraordinary ability to induce expression of endogenous, multiple enzymes via the upregulation of Nrf2 function [24]. Our previous study has found that SFN had a beneficial effect on type 1 diabetic vascular complications [5]. However, there was no report yet whether SFN can prevent the development of aortic pathogenesis alterations in T2DM.

**Figure 1:**
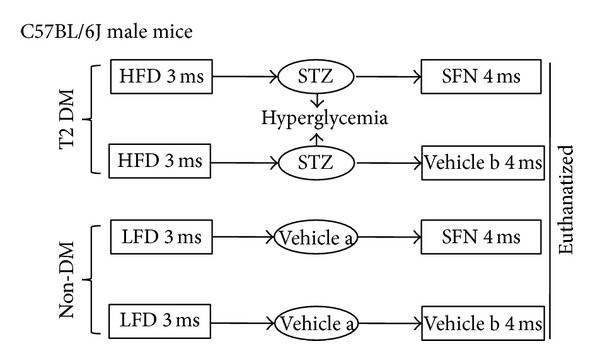
Schematic illustration for the animal experimental design.

To this end, we used a type 2 diabetic mouse model to verify the protective function of SFN against diabetic aortic damage and dissect the underlying mechanisms. The experimental design was illustrated in Figure 1. Type 2 diabetic and age-matched control mice were treated with SFN for 4 months. At the end of 4 months treatment of SFN mice were euthanatized for collecting tissues to perform the experimental measurements.

## 2. Materials and Methods

### 2.1. Animals

C57BL/6J male mice, 8–10 weeks of age, were purchased from the Jackson Laboratory (Bar Harbor, Maine) and housed at the University of Louisville Research Resources Center at 22°C with a 12 h light/dark cycle with free access to food and tap water. All experimental procedures for these animals were approved by the Institutional Animal Care and Use Committee of the University of Louisville, which is compliant with the Guide for the Care and Use of Laboratory Animals published by the US National Institutes of Health (NIH Publication number 85–23, revised 1996).

### 2.2. Type 2 Diabetes Model

To establish type 2 diabetic model, mice were fed with high-fat diet (HFD, Research Diets 12492, 60% kcal from fat) for 3 months, which made these mice become significantly obese (44.6 ± 2.9 g for HFD versus 32.4 ± 1.5 g for control, *P* < 0.05). Insulin resistance was also induced, shown by the increased area size under curves for both *Intraperitoneal (IP) glucose tolerance test (IPGTT) *(35493.28 ± 5270.90 for HFD versus 25114.82 ± 4630.55 for control, *P* < 0.05) and *IP insulin tolerance test (IPITT)* (15403.26 ± 3252.76 for HFD versus 9790.5 ± 3462.36 for control, *P* < 0.05). These insulin resistant mice were randomly injected intraperitoneally with STZ (Sigma-Aldich, St. Louis, MO, dissolved in 0.1 M sodium citrate (pH4.5, Vehicle a, Figure 1)) at 100 mg/kg body weight once [25, 26]. Five days after STZ injection, mice with hyperglycemia (blood glucose levels ≥ 250 mg/dL, blood sample collected from the tail vein measured using a Free Style Lite glucometer (Abbott Diabetes Care, Alameda, CA)) were defined as diabetic. In parallel, age-matched control mice were given low-fat diet (LFD, Research Diets 12450B, 10% kcal from fat) for 3 month, followed by an injection of the same volume of sodium citrate buffer when HFD-fed mice were received STZ injection. Both diabetic and control mice continually received HFD or LFD feeding for additional 4 months (Figure 1). During this 4-month period, both diabetic and control mice were further divided into two groups, with and without SFN treatment (Sigma-Aldich), which were given SFN at 0.5 mg/kg subcutaneously for five days per week. Dose of SFN used was based on our previous study [27].

At the end of the additional 4 months, these diabetic mice remained showing the significantly increased insulin resistance (29230 ± 3173.43 for HFD versus 7947.50 ± 1209.02 for control, *P* < 0.05) and blood glucose (324.66 ± 51.07 for HFD versus 116.00 ± 11.04 for control, *P* < 0.05), insulin (0.8293 ± 0.13 for HFD versus 0.55 ± 0.05 for control, *P* < 0.05), triglyceride (395.05 ± 64.43 for HFD versus 48.48 ± 4.80 for control, *P* < 0.05), and cholesterol (139.12 ± 6.53 for HFD versus 80.39 ± 15.56 for control, *P* < 0.05) levels; all of which are typical magnifications, suggesting the induction of T2DM. In summary, there were four groups of mice (*n* = 6 at least per group): LFD control (Ctrl), LFD/SFN (SFN), type 2 diabetes (DM), and DM plus SFN (DM/SFN). At the end of 4 months of SFN treatment, mice were euthanatized for experimental measurements. Since SFN was dissolved in 1% dimethyl sulfoxide (DMSO) and diluted in PBS, mice serving as controls were also subcutaneously given the same volume of PBS containing 1% DMSO (Vehicle b, Figure 1), based on our own and other studies [27–29].

### 2.3. Aorta Preparation and Histopathological Examination

After mice were anesthetized with 2,2,2-tribromoethanol (commercial name: avertin) at 4–6 mg/kg body weight, thoraxes were opened and the descending thoracic aortas were isolated carefully without rips or cuts. Aortic tissues were fixed in 10% buffered formalin overnight. The fixed tissues were cut into ringed segments (approx. 2-3 mm in length) so they can be dehydrated in graded alcohol series, clean with xylene, embedded in paraffin, and sectioned at 5 *μ*m thickness for pathological and immunohistochemical or immunofluorescent staining. Histological evaluation of aorta was performed by H&E staining. The thickness of aorta was evaluated by measuring the width of tunica media using Image Pro Plus 6.0 software. For immunohistochemical or immunofluorescent staining, paraffin sections from aortic tissues were dewaxed, incubated with 1x Target Retrieval Solution (Dako, Carpinteria, CA) in a microwave oven for 15 min at 98°C for antigen retrieval, followed by 3% hydrogen peroxide for 10 min at room temperature and 5% bovine serum albumin for 60 min, respectively. These sections were then incubated with primary antibodies against connective tissue growth factor (CTGF) at 1 : 100 dilution (BD Bioscience, San Jose, CA) and transforming growth factor (TGF-*β*1) at 1 : 100 dilution (Santa Cruz Biotechnology, Santa Cruz, CA, USA), tumor necrosis factor-alpha (TNF-*α*) at 1 : 50 dilution (Abcam, Cambridge, MA), vascular cell adhesion molecule 1 (VCAM-1) at 1 : 100 dilution (Santa Cruz Biotechnology, Santa Cruz, CA, USA), 3-nitrotyrosine (3-NT) at 1 : 400 dilution (Millipore, Billerica, CA), 4-hydroxy-2-nonenal (4-HNE) at 1 : 400 dilution (Alpha Diagnostic International, San Antonio, TX), Nrf2 at 1 : 50 dilution, and Cu-Zn super oxide dismutase-1 (SOD-1) at 1 : 400 dilution (both from Santa Cruz Biotechnology, Santa Cruz, CA, USA) over night at 4°C. Afterwards sections were washed with PBS, and incubated with horseradish peroxidase conjugated secondary antibody (1 : 100–400 dilutions with PBS) or Cy3-coupled donkey anti-rabbit or anti-goat IgG secondary antibody (1 : 200 dilution with PBS) for 1 h at room temperature. For color development purposes, immunohistochemical staining sections were treated with peroxidase substrate DAB kit (Vector Laboratories, Inc. Burlingame, CA) and counterstained the nuclei with hematoxylin, while immunofluorescent staining sections were stained with DAPI at 1 : 1000 dilution to localize the nucleus.

For quantitative analysis of these immunohistochemical and immunofluorescent staining, the Nikon Eclipse E600 microscopy system was used and 3 sections at interval of 10 sections from each aorta (per mouse) were selected and at least five high-power fields randomly sections were randomly recaptured. Image Pro Plus 6.0 software was used to translate the interesting area staining density into an integrated optical density (IOD) that was divided by the area size of interest to reflect the staining intensity, and the ratio of IOD/area size in experimental group was presented as a fold relative to that of control.

### 2.4. Sirius-Red Staining for Collagen

Aortic fibrosis was detected by Sirius-red staining of collagen, as described in our previous study [6]. Briefly sections were stained with 0.1% Sirius-red F3BA and 0.25% Fast Green FCF. The stained sections were then assessed for the presence of collagen using a Nikon Eclipse E600 microscopy system.

### 2.5. Terminal Deoxynucleotidyl-Transferase-Mediated dUTP Nick-End Labeling (TUNEL) Staining

TUNEL staining was performed with formalin-fixed, paraffin-embedded sections using peroxidase *in situ* Apoptosis Detection Kit S7100 (Millipore, Billerica, MA), according to the manufacture's instruction. The positively stained apoptotic cells were counted randomly in five microscopic fields at least for each of the three slides from each mouse under light microscopy. The percentage of TUNEL positive cells relative to 100 nuclei was presented.

### 2.6. Proliferating Cell Nuclear Antigen (PCNA) Staining

The PCNA staining kit (Invitrogen, Camarillo, CA) was used for staining proliferating cells, according to the manufacture's instruction. The positively stained proliferating cells were counted randomly in five microscopic fields at least for each of the three slides per mouse under light microscopy. The percentage of PCNA positive cells relative to 100 nuclei was presented.

### 2.7. Real-Time qPCR

Aortas were frozen with liquid nitrogen and stored at −80°C. Total RNA was extracted using the TRIzol Reagent (Invitrogen, USA). RNA concentrations and purity were quantified using a Nanodrop ND-1000 spectrophotometer. First-strand complimentary DNA (cDNA) was synthesized from total RNA according to manufacturer's protocol from the RNA PCR kit (Promega, Madison, WI). Reverse transcription was performed using 0.5 *μ*g of total RNA in 12.5 *μ*L of the solution containing 4 *μ*L 25 mM MgCl_2_, 4 *μ*L AMV reverse transcriptase 5x buffer, 2 *μ*L dNTP, 0.5 *μ*L RNase inhibitor, 1 *μ*L of AMV reverse transcriptase, and 1 *μ*L of oligodT primer, which were added with nuclease-free water to make a final volume of 20 *μ*L. Reaction system was run at 42°C for 50 min and 95°C for 5 min. Primers of Nrf2, SOD-1, HO-1, and GAPDH were purchased from Applied Biosystems (Carlsbad, CA). Real-time quantitative PCR (qPCR) was carried out in a 20 *μ*L reaction buffer that included 10 *μ*L of TaqMan Universal PCR Master Mix, 1 *μ*L of primer, and 9 *μ*L of cDNA with the ABI 7300 Real-Time PCR system. The fluorescence intensity of each sample was measured at each temperature change to monitor amplification of the target gene. The comparative cycle time (CT) was used to determine fold differences between samples.

### 2.8. Statistical Analysis

Data were presented as mean ± SD (*n* = 6). Comparisons were performed by two-way ANOVA for the different groups. When there was significant difference among groups, the repetitive comparing Tukey's test was used to further analyse with Origin 7.5 Lab data analysis and graphing software. *P* < 0.05 was considered as statistical significance.

## 3. Result

### 3.1. SFN Prevented T2DM-Induced Aortic Pathological Changes and Fibrosis

**Figure 2:**
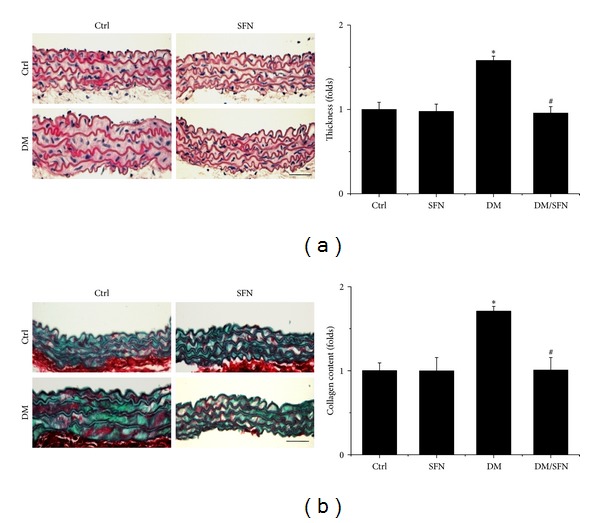
Protective effect of SFN on diabetes-induced aortic pathological changes. The pathogenic changes of aortas were examined by H&E staining (a) and the accumulation of collagen was detected by Sirius-red staining (b), followed by semiquantitative analysis. Data were presented as means ± SD (*n* = 6); *P < 0.05 versus corresponding Ctrl; ^#^ *P* < 0.05 versus corresponding DM. Bar = 50 *μ*M.

At the end of experiment, aortas were examined pathologically by H&E staining, which displayed significantly increase of the tunica media thickness in T2DM mice (Figure 2(a)). Sirius-red staining also revealed an increased collagen accumulation in tunica media of aortas in T2DM group (Figure 2(b)). However, all these pathological changes observed in the aortas of T2DM mice were completely prevented by the 4-month SFN treatment.

**Figure 3:**
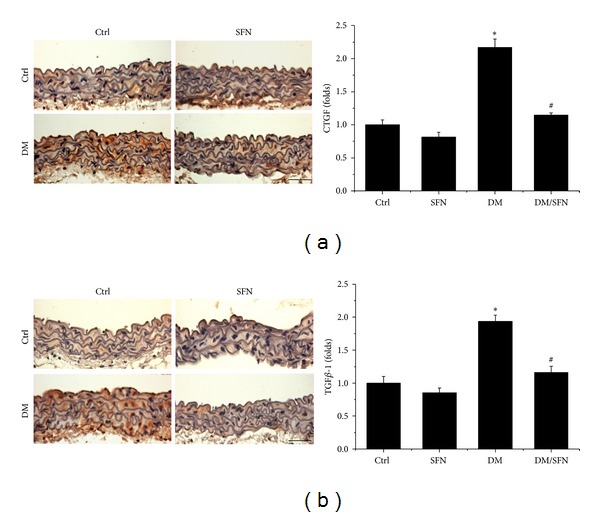
Protective effect of SFN on diabetes-induced aortic fibrosis. Aortic fibrosis was examined by immunohistochemical staining for the expression of CTGF (a) and TGF-*β*1 (b), followed by semiquantitative analysis. Data were presented as means ± SD (*n* = 6); *P < 0.05 versus corresponding Ctrl; ^#^ *P* < 0.05 versus corresponding DM. Bar = 50 *μ*M.

To further detect the preventive effect of SFN on T2DM-induced aortic fibrosis, immunohistochemical staining showed the increased expression of profibrotic mediators, CTGF (Figure 3(a)), and TGF-*β*1 (Figure 3(b)), in aortic tunica media of diabetic mice. Supplementation with SFN completely prevented these fibrotic responses in the aortas of type 2 diabetic plus SFN mice (DM/SFN group).

### 3.2. SFN Prevented T2DM-Induced Aortic Inflammation and Oxidative Damage

**Figure 4:**
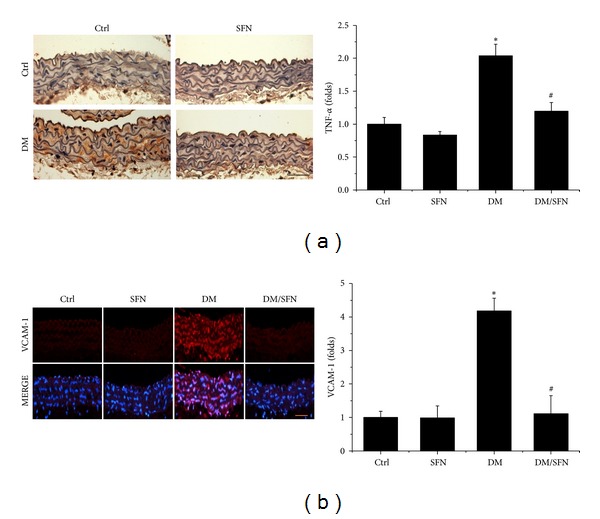
Protective effect of SFN on diabetes-induced aortic inflammation. Aortic inflammation was examined by immunohistochemical staining for the expression of TNF-*α* (a) and immunofluorescent staining for the expression of VCAM-1 (red) (b), followed by semiquantitative analysis. Data were presented as means ± SD (*n* = 6); *P < 0.05 versus corresponding Ctrl; ^#^ *P* < 0.05 versus corresponding DM. Bar = 50 *μ*M.

On account of the fact that both inflammation and oxidative damage are primary risk factors for the vascular endothelium remodeling, the expression of TNF-*α* (Figure 4(a)) and VCAM-1 (Figure 4(b)) was examined with immunohistochemical and immunofluorescent staining, which showed a significant increase in aortic tunica media of T2DM mice, an effect that was completely prevented by 4-month SFN treatment.

**Figure 5:**
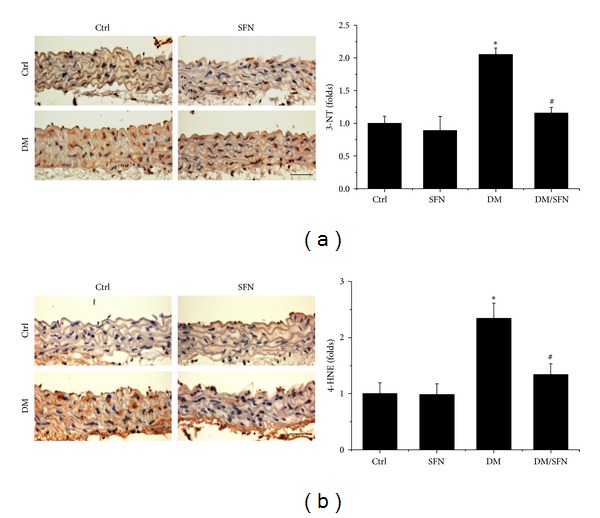
Protective effect of SFN on diabetes-induced aortic oxidative damage. Aortic oxidative damage was examined by immunohistochemical staining for the accumulation of 3-NT (a) and 4-HNE (b), followed by semiquantitative analysis. Data were presented as means ± SD (*n* = 6); *P < 0.05 versus corresponding Ctrl; ^#^ *P* < 0.05 versus corresponding DM. Bar = 50 *μ*M.

Considering that inflammation and oxidative stress are reciprocal cause and outcomes, oxidative and nitrative damage was examined by immunohistochemical staining for increased accumulation of 3-NT (Figure 5(a)) and 4-HNE (Figure 5(b)), which was found to be significantly increased in the aortic tunica media of T2DM mice. However, treatment with SFN for 4 months completely prevented the oxidative damage (Figures 5(a) and 5(b)).

### 3.3. SFN Prevented T2DM-Induced Aortic Apoptotic Cell Death and Proliferation

**Figure 6:**
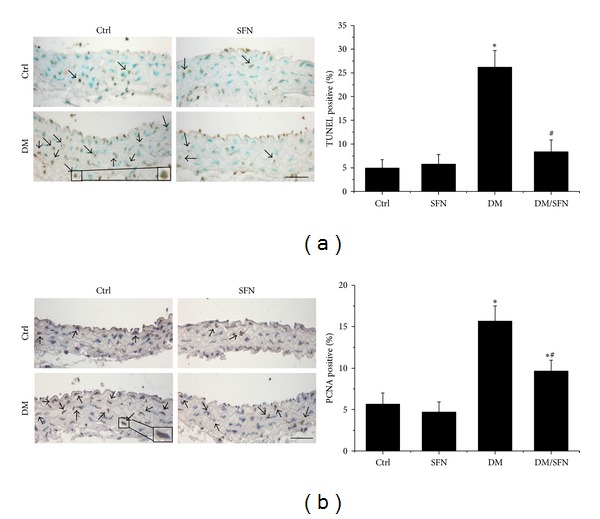
Diabetes-induced aortic apoptosis and proliferation increased. The apoptotic cell was examined by TUNEL staining (a) and the proliferation of aortic tunica media was examined by PCNA staining (b), followed by semiquantitative analysis. Data were presented as means ± SD (*n* = 6). *P < 0.05 versus corresponding Ctrl; ^#^ *P* < 0.05 versus corresponding DM. Bar = 50 *μ*M.

We recently reported the induction of apoptotic cell death and cell proliferation in the aortas of T2DM mice [6]. The next study was conducted to further examine the effect of SFN on the cell death and proliferation by TUNEL staining (Figure 6(a)) and PCNA staining (Figure 6(b)), which showed significant increases of apoptotic cell death and proliferation in the aortas of T2DM mice, but not in the aortas of diabetic mice with SFN administration (DM/SFN group).

### 3.4. SFN Upregulated the Expression of Nrf2 and Its Downstream Genes

**Figure 7:**
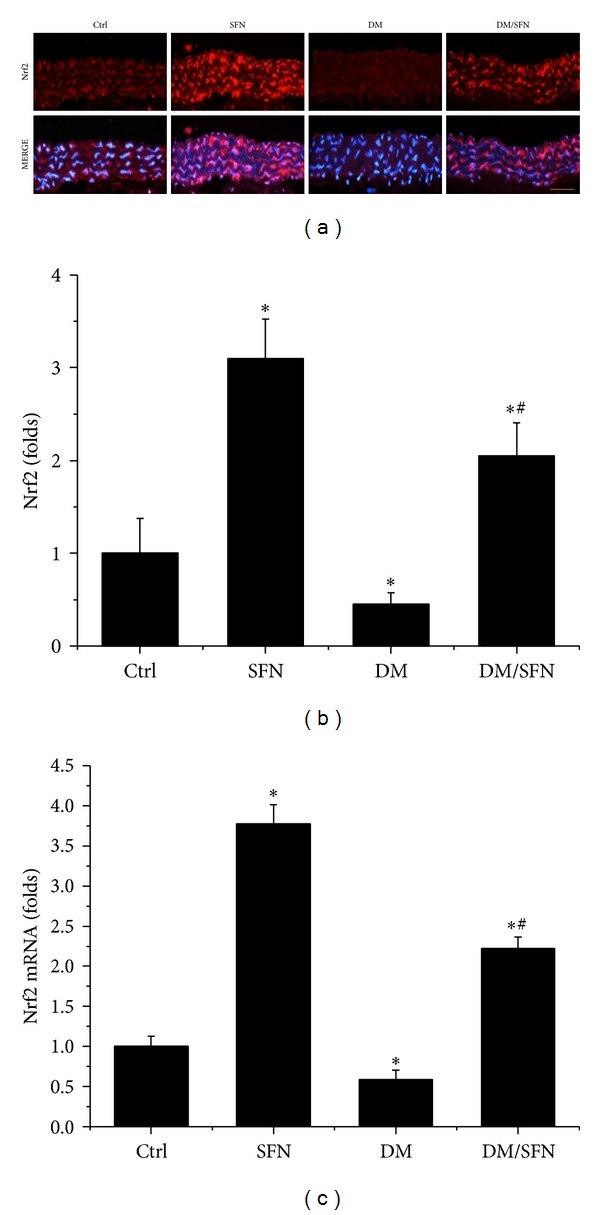
Effects of SFN on aortic expression of Nrf2. Aortic expression of Nrf2 was examined by immunofluorescent staining for its protein expression (red) (a) with semiquantitative analysis (b) and real-time PCR for its mRNA level (c). Data were presented as means ± SD (*n* = 6). *P < 0.05 versus corresponding Ctrl; ^#^ *P* < 0.05 versus corresponding DM. Bar = 50 *μ*M.

**Figure 8:**
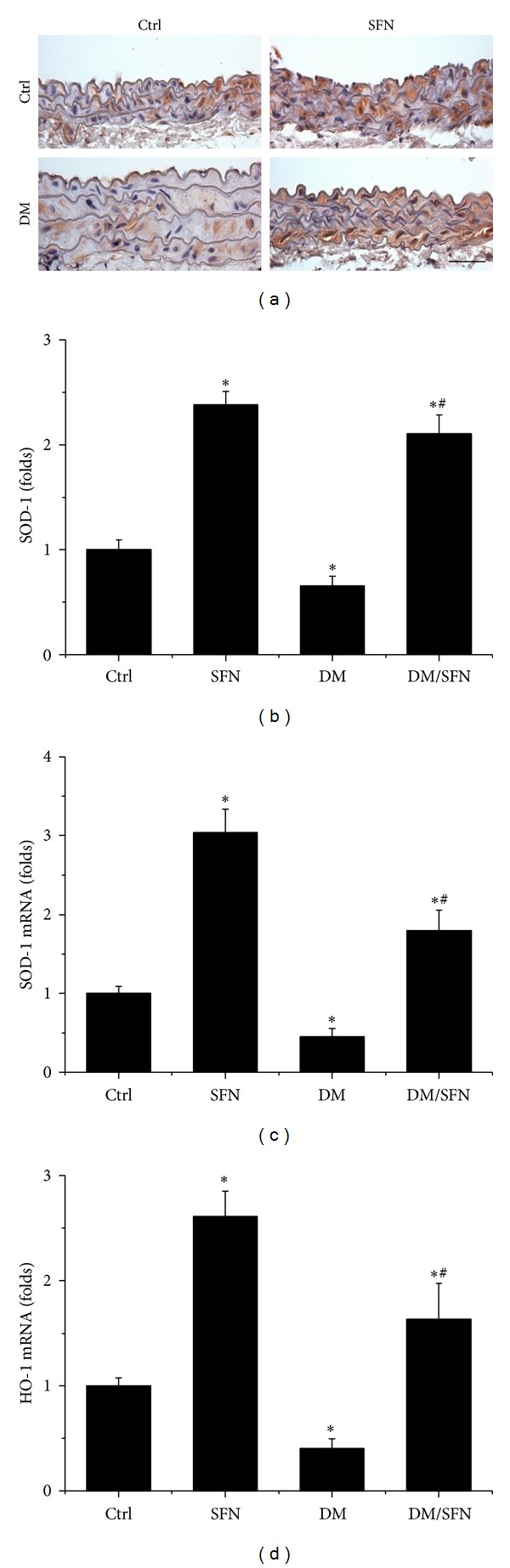
Effects of SFN on aortic expression of Nrf2 downstream genes. Aortic expression of Nrf2 downstream genes SOD-1 expression was examined by immunohistochemical staining for protein expression (a) in aortic tunica media with semiquantitative analysis (b) and real-time PCR at mRNA level SOD-1 (c) and HO-1 (d). Data were presented as means ± SD (*n* = 6). *P < 0.05 versus corresponding Ctrl; ^#^ *P* < 0.05 versus corresponding DM. Bar = 50 *μ*M.

The above results showed that SFN protected diabetic induction of aortic fibrosis, inflammation, and oxidative damage. Considering that oxidative stress has been extensively considered as the pivotal mediator for various cardiovascular complications of diabetic patients, we assume that the above pathological changes in the aortas of T2DM mice may predominantly attribute to the increased oxidative stress. The protective effect of SFN on diabetes-induced aortic pathogenesis may be mediated by upregulation of endogenous antioxidants. SFN is an Nrf2 activator [21], therefore, whether SFN protects the aorta from diabetes by activating Nrf2 was examined first by measuring the expression and transcription of Nrf2. Immunofluorescent staining showed that diabetes significantly decreased Nrf2 protein (Figures 7(a) and 7(b)) expression in the aorta of T2DM compared to control. Similarly, the Nrf2-downstream gene SOD-1 protein (Figures 8(a) and 8(b)) expression also decreased in the aorta of T2DM compared to control. Furthermore, diabetes also significantly downregulated the mRNA expression of Nrf2 (Figure 7(c)) and its downstream antioxidant genes SOD-1 (Figure 8(c)) and HO-1 (Figure 8(d)) in the aorta of T2DM compared to control. Although 4 months of SFN treatment significantly increased Nrf2 and its downstream antioxidant genes at both protein and mRNA levels in non-DM and T2DM mice.

## 4. Discussion

We have provided the first experimental evidence to show the significant protective effect of SFN on the aorta against T2DM-induced damage. Significant increase of aortic wall thickness, fibrosis, inflammation, oxidative damage, apoptosis and proliferation was developed in type 2 diabetic mice, and these pathological changes were significantly prevented by treatment with SFN, which is associated with the upregulation of aortic Nrf2 expression and transcription.

Chronic inflammation plays an important role for the development of various chronic pathogeneses, including diabetes [30–33]. The effects of chronic inflammation include induction of oxidative stress, apoptotic cell death, and abnormal cell proliferation, all of which could contribute to the tissue structural and functional abnormalities [30–33]. In the present study we demonstrated the diabetic induction of aortic inflammation, shown by increased expression of TNF-*α* (Figure 4(a)), VACM-1 (Figure 4(b)) in the aorta of T2DM, which was accompanied with increased aortic oxidative stress (3-NT (Figure 5(a)) and 4-HNE (Figure 5(b))), apoptotic cell death (TUNEL (Figure 6(a))), cell proliferation (PCNA (Figure 6(b))), and remodeling (CTGF (Figure 3(a)) and TGF-*β*1 (Figure 3(b))) in T2DM group. All these pathogenic alterations were prevented by SFN administration. These findings are consistent with the classic concept that inflammation and oxidative stress are reciprocal cause and outcomes [7], both of which are main pathogenic factors for the development of various cardiovascular diseases under stress conditions.

It is known that Nrf2 expression and transcription *in vitro* and *in vivo* are increased in response to oxidative stress [34–36]. Ungvari et al. found that HFD increased endothelia ROS levels and endothelial dysfunctions were significantly severer in Nrf2-KO mice than in wild-type mice [37], indicating that adaptive upregulation of Nrf2-driven antioxidant systems effectively attenuates cellular oxidative stress under diabetic conditions [37]. There are a few studies recently, indicating the protective effect by activation of Nrf2 with various compounds on the aortas under various pathological conditions [6, 38, 39]. Here we have found that SFN treatment also significantly increased Nrf2 expression and function (Figure 7), which indicates that SFN prevents diabetes-induced aortic pathogenesis that may be associated with the upregulation of Nrf2.

In previous studies from our group and others, Nrf2 was found to play a critical role in preventing diabetes-induced aortic damage [5, 6, 19] and cardiac or renal damage [40–43]. We found that Nrf2 expression in the aorta was significantly upregulated at 3 months without significant aortic damage, but significantly downregulated at 6 months along with significant aortic damage in type 1 diabetic mouse model [5]. Similarly, aortic expression of Nrf2-driven antioxidant enzymes markedly increases in young mice fed a HFD, but tend to decrease or only mild increase in middle-aged mice fed a HFD, despite the fact that vascular oxidative stress is more severe in HFD-fed middle-aged mice than in young mice [44]. Consistent with these previous studies, we found the significant reduction of Nrf2 expression at both protein and mRNA levels in the aorta of type 2 diabetic mice at 4 months (Figure 7), along with significant downregulation of its transcription function that is reflected by the expression of its downstream antioxidant genes SOD-1 and HO-1 (Figure 8), which were accompanied by significant aortic damage. Most importantly, SFN-treated type 2 diabetic mice showed a significant increase of aortic Nrf2 expression and function. The upregulated Nrf2 and its downstream antioxidant genes in SFN-treated type 2 diabetic mice efficiently reduced diabetes-induced oxidative damage, inflammation, apoptosis, proliferation, and remodeling and eventually significantly prevented the aortic pathological and structural changes.

## 5. Conclusions

In summary, to our knowledge, this is the first study to investigate the protective effects of SFN against T2DM-induced aortic pathological changes. We found that treatment with SFN can completely reverse and/or prevent the progression of diabetes-induced aortic fibrosis, inflammation, oxidative damage, apoptosis, and proliferation in T2DM mice. Mechanism responsible for the preventive effect of SFN is related to upregulation of Nrf2 expression and function to afford potent antioxidant effect. Considering the fact that SFN is a molecule able to be obtained from cruciferous vegetables such as broccoli, cauliflower, or cabbages [24], our findings would be very important for patients with T2DM consider intaking foods rich with SFN for preventing vascular complications.
